# Hand-Work, Head-Work, and Symmetry of Brains

**Published:** 1879-09

**Authors:** 


*Head-work, Hand-work, and Symmetry of Brains.*—Measurements ranging through a diversity of subjects demonstrates that students have the left frontal region of the head more largely developed than the right; while the illiterate have the right occipital most largely developed. The dentist, working both hand and brain, should be of symmetrical brains, surely. H.

